# Effect of iodine supplementation during pregnancy on neonatal thyroid stimulating hormone

**DOI:** 10.1186/1687-9856-2013-S1-P153

**Published:** 2013-10-03

**Authors:** Dabuswinee Sukkhojaiwaratkul, Pat Mahachoklertwattana, Preamrudee Poomthavorn

**Affiliations:** 1 Department of Pediatrics, Faculty of Medicine Ramathibodi Hospital, Mahidol University, Bangkok 10400, Thailand

## Background

Thyroid hormone is essential for neurological development, particularly in the developing brain. Iodine is the important constituent of thyroid hormone. Its requirement during pregnancy is increased. Elevated neonatal thyroid stimulating hormone (TSH) is a sensitive indicator of iodine insufficiency in pregnant women. Our previous study in the year 2000 demonstrated that median urine iodine (UI) concentration in pregnant women was 85 µg/L, a level of which indicates mild iodine deficiency. Since October 2010, Thai national universal iodine supplementation program has been started by prescribing a 150 µg-iodine tablet daily to all pregnant women throughout their gestation. We, therefore, aimed to determine the effect of maternal iodine supplementation on neonatal TSH.

## Study design

Retrospective data collection.

## Methods

Cord blood TSH measurement for screening of congenital hypothyroidism has been routinely performed at Ramathibodi Hospital, Mahidol University, Bangkok, Thailand. Data of neonatal TSH during the years 2007-2011 were collected. Neonates born before October 2010 were classified as pre-iodine supplementation group (Group 1) while those born after that were post-iodine supplementation group (Group 2).

## Results

Cord blood TSH values were obtained from 6,802 neonates, 4,864 in Group 1 and 1,938 in Group 2. Median (range) neonatal TSH of Group 1 was significantly higher than that of Group 2 [7.4 (0.01-87.7) vs. 5.2 (0.7-35.1)] mU/L, respectively (p <0.001). Eighty-one percent of neonates in Group 1 but only 54% in Group 2 had cord blood TSH of greater than 5 mU/L. Similarly, 26% of neonates in Group 1 but only 13% in Group 2 had cord blood TSH of greater than 10 mU/L.

## Conclusions

There was a trend towards declining of cord blood TSH values after iodine supplementation during pregnancy. This finding suggests that mothers residing in Bangkok are likely to have mild iodine deficiency. Median neonatal TSH value is useful for monitoring iodine nutrition in the population.

